# Prognostic implication of human papillomavirus types in cervical cancer patients: a systematic review and meta-analysis

**DOI:** 10.1186/s13027-020-00332-5

**Published:** 2020-11-07

**Authors:** Yuanyuan Xu, Yichao Qiu, Shuang Yuan, Hongjing Wang

**Affiliations:** 1 Department of Obstetrics and Gynecology, West China Second Key Laboratory of Birth Defects and Related Diseases of Women and Children (Sichuan University), Ministry of Education, West China Second University Hospital, Sichuan University, Chengdu, China; 2 Department of Obstetrics and Gynecology, West China Second University Hospital, Sichuan University, No.20, Section 3, South People’s Road, Chengdu, Sichuan P.R. China

**Keywords:** Human papillomavirus 16, Human papillomavirus 18, Survival, Cervical cancer

## Abstract

**Background:**

To estimate the prognostic relevance of human papillomavirus (HPV) 16 and HPV 18 in patients with cervical cancer.

**Method:**

We searched PubMed, EMBASE, American Society of Clinical Oncology (ASCO) and the European Society of Medical Oncology (ESMO), CNKI, and Wanfang databases to search primary articles illustrating the survival outcomes in cervical cancer patients with or without HPV 16/18 infection. A meta-analysis was conducted to generate a combined hazard ratio (HR) with 95% confidence intervals (CI) for progression-free survival (PFS), disease free survival (DFS) and overall survival (OS).

**Results:**

A total of 13 studies were included. Our meta-analysis revealed that HPV 16 positive did not have any impact on OS (HR, 0.76; 95% CI = 0.37–1.54; *P* = 0.44). Cervical cancer patiensts infected with HPV 18 had worse OS (HR, 1.66; 95% CI = 1.28–2.17; *P* = 0.0001), DFS (HR, 2.10; 95% CI = 1.73–2.54; *P* < 0.0001) and worse PFS (HR, 2.97; 95% CI = 1.69–5.23; *P* = 0.00012) compared with those not infected with HPV 18. cervical cancer patiensts infected with HPV 18 had worse PFS compared with those infected with HPV 16 ((HR, 1.34; 95% CI = 1.06–1.70; *P* = 0.01).

**Conclusion:**

Cervical cancer patients infected with HPV 18 had worse survival compared with cervical cancer patients with HPV 16 infection.

## Background

Cervical cancer occurs in the cells of the cervix, with highest incidence rates found among women between 40 and 60 years old. Cervical cancer is the second most common female cancer worldwide [1]. The prevalance of cervical cancer is 4640 per 100,000 people according to the European standard population, with a relative 5-year-survival rates of 68% [2]. Approximately twelve high risk genotypes of the human papillomavirus (HPV) play a role in the development of cervical cancer. Nowdays, many countries have introduced HPV vaccine to reduce HPV infection and the risk to develop cervical cancer. HPV 16 and HPV 18 are the most common genotypes identified in cervical carcinoma, representing 70% of all infections [3]. Among these two genotypes, HPV 16 is the most common genotype worldwide found in patients with invasive cervical cancer. The prevalance of HPV 16 infection ranged from 42 to 75% [4–8], followed by HPV 18 with a prevalance of 10 to 30% [4, 8–13]. HPV 16 and HPV 18 are among the genotypes (HPV 16, 18, 31, 33, 35, 45, 52 and 58) which are strongly associated with progression to cervical cancer compared with other high risk genotypes and low risks genotypes [14, 15]. High risk genotypes include HPV 16, 18, 31, 33, 35 39, 45, 51, 52, 56, 58, 59, 68, 73 and 82. Low risk genotypes include HPV 6, 11, 40, 42, 43, 44, 54, 61, 70, 72 and 81, which are associated with benign lesions [16, 17].

Several factors would impact the prognosis for cervical cancer patients, such as tumor staging, size and metastatis. Although the effect of HPV on the development of cervical cancer has been well eastablished, its function of prognosis is still not well understood. Several studies have found the prognosis role of HPV 18 infections in early stage cervical cancer patients [10, 11, 18]. Several studies have found that patients diagnosed with early stage cervical cancer and infected by HPV 18 have worse prognosis. A retrospective study including 116 cervical cancer patients which received primary surgical treatment showed that positivity for HPV 18 was associated with shorter progression free survival (PFS) (HR: 5.2, 95% CI = 1.29–20.9, *P* = 0.02) [8]. Another population-based study with 24,041 women also found that HPV 18 infection was an independent prognostic factor for 3-year survival in cervical cancer (HR: 1.704, 95% CI = 1.095–2.654) [13]. However, controversy still exists regarding the prognostic relevance of HPV 18 in patients with cervical cancer. A study with 236 stage I-III Chinese cervical cancer patients aged 26 to 87 years after receiving primary treatment proved that HPV 18 did not have significant impact on disease free survival (DFS) (hazard ratio (HR): 1.49, 95% confidence interval (CI) = 0.78–2.86) or overall survival (OS) (HR: 1.23, 95% CI = 0.66–2.27) [4]. A brazil cohort study conducted with 86 stage I cervical cancer patients found that the presence of HPV 18 would not affect DFS (HR: 0.797, 95% CI = 0.175–3.640) [9].

The prognostic impact of HPV 16 on survival in patients with cervical cancer is also controversial [4–7, 9]. Although these studies suggested no significant impact of HPV infection on DFS or PFS, its significance on OS did not reach consistency. A Chinese study consisting 306 cervical cancer patients found that presence of HPV 18 was negatively associated with OS (HR: 0.36, 95% CI = 0.18–0.74, *P* = 0.005) [5]. A Korea study consisting 298 patients I-V stage cervical cancer patients also proved the significant relevance of HPV 18 on OS (HR: 0.558, 95% CI = 0.326–0.955, *P* = 0.033) [6]. However, other studies proved that HPV 16 was not a prognoctic factor for OS in cervical cancer patients. Yat Ming Lau and collegues found that presence of HPV 18 was not significant for OS in patients with stage I-III cervical cancer (HR: 0.99, 95% CI = 0.64–1.55). A Japanese study consisting 137 stage I-IV cervical cancer also found no survival relevance of HPV 18 infection (HR: 0.42, 95% CI = 0.15–1.04, *P* = 0.06) [7]. However, to date, no previous study published the systematic review and meta analysis of impact of HPV on survival in cervical cancer patients.

Thus, we performed an updated systematic review and meta-analysis to summarize the impact of HPV 16 and HPV 18 on survival in patients with cervical cancer.

## Methods

### Literature search

PubMed, EMBASE, American Society of Clinical Oncology (ASCO) and the European Society of Medical Oncology (ESMO), CNKI, and Wanfang databases were searched by our researchers using common keywords related to HPV 16, HPV 18, cervical cancer and survival. The following keywords were included: human papillomavirus 16, human papillomavirus 18, cervical cancer, DFS, PFS and OS. We reviewed the details of these relevant publications for additional papers.

### Selection criteria

We selected articles that met the following criteria: (1) the clinical study recruited patients with pathologically or cytologically diagnosed cervical cancer; (2) the clinical study investigated survival related results, such as PFS, DFS and OS with a HR and 95% CI.

### Study results extraction

Two independent researchers in our department read all the publications independently and discussed the study extraction until they reached consensus. The criterias defined by Cochrane Handbook for Systematic Reviews of Interventions version 5.1.0 were used [19], the following six domains were assessed: (1) randomization generation, (2) allocation concealment, (3) participants and personnel blinding, (4) outcome assessment blinding, (5) incomplete outcome data, (6) reporting selective outcome. Data obtained from the studies included the author, year of publication, patient source (region), age of patients, number of patients and survival outcomes.

### Statistical analysis

We chose PFS, DFS and OS as the endpoints in our meta-analysis. The survival data associated with HPV 16 and HPV 18 were summarized in Tables 1, 2 and 3. HR and 95% CI were used as measures of the prognostic value using Review Manager (RevMan) Version 5.4, for Windows. Publication bias was evaluated according to the funnel plot and Begg’s and Egger’s tests. Statistical heterogeneity was calculated using the Chi-square test and also calculation of the I^2^ statistic. We considered an I^2^ value > 50% to indicate a significant heterogeneity between these studies. A random effects model was used if significant heterogeneity was detected among studies. If I^2^ value was below 50%, results were measured using a fixed effects model.

**Table 1:** Study characteristics of studies investigating the prognostic relevance of HPV-16

Author	Year	Country	Total number of patients	Mean age	Clinical stage	Treatment	Number of patients with HPV-16 positive	Number of patients with HPV-18 negative	Median DFS	Median PFS	Median OS
Yat Ming Lau	2015	Hong Kong, China	236	54.4	I-III	radiotherapy+/−chemotherapy	142	94	73.2% vs. 81.2%, HR: 1.54, 95% CI = 0.93–2.56	NA	71.6% vs. 81.7%, HR: 0.99, 95% CI = 0.64–1.55
Dong Hang	2017	China	306	48	I-IV	surgery alone, surgery plus adjunctive chemotherapy, radiotherapy or chemoradiotherapy, concurrent chemoradiotherapy, chemotherapy or radiotherapy only.	186	120	NA	NA	HR: 0.36, 95% CI = 0.18–0.74, *P* = 0.005
Mamiko Onuki	2018	Japan	137	49.2	I-IV	Surgery+radiotherapy	59	78	NA	NA	HR: 0.42, 95% CI = 0.15–1.04, *P* = 0.06
Byoung Hyuck Kim	2019	Korea	298	48	I-IV	radiotherapy	164	127	NA	77.6 vs. 57.7%, *P* = 0.022	HR: 0.558, 95% CI = 0.326–0.955, *P* = 0.033
Sun-Hye Yang	2014	Korea	116	NA	I-IIA	surgery	49	67	NA	HR: 1.33, 95% CI = 0.31–5.67, *P* = 0.70	Not significant
Rossana de Arau ´jo Cata ~o Zampronha	2013	Brazil	86	40	I	Surgery+radiotherapy	30	56	HR: 1.104, 95% CI = 0.243–5.007	NA	NA

*HPV* Human papillomavirus, *DFS* Disease free survival, *HR* Hazard ratio, *CI* Confidence interval, *NA* Not available, *DFS* Disease free survival, *PFS* Progression free survival, *OS* Overall survival

**Table 2:** Study characteristics of studies investigating the prognostic relevance of HPV-18

Author	Year	Country	Total number of patients	Mean age	Clinical stage	Treatment	Number of patients with HPV-18 positive	Number of patients with HPV-18 negative	Median DFS	Median PFS	Median OS
Yat Ming Lau	2015	Hong Kong, China	236	54.4	I-III	radiotherapy+/−chemotherapy.	30	185	73.2% vs. 78.2%, HR: 1.49, 95% CI = 0.78–2.86	NA	80% vs. 75.7%, HR: 1.23, 95% CI = 0.66–2.27
Sun-Hye Yang	2014	Korea	116	NA	I-IIA	surgery	15	101	NA	HR: 5.2, 95% CI = 1.29–20.9, *P* = 0.02	Not significant
Rossana de Arau ´jo Cata ~o Zampronha	2013	Brazil	86	40	I	Surgery+radiotherapy	25	51	HR: 0.797, 95% CI = 0.175–3.640	NA	NA
Robert A. Burger	1996	USA	291	NA	I-IV	radical hysterectomy and pelvic lymphadenectomy	58	233	NA	NA	HR: 2.59, 95% CI = 1.08–6.22
Chyong-Huey Lai	2007	Taiwan, China	1067	50	IA-IIA	surgery	176	891	HR: 1.8, 95% CI = 1.8–2.7		HR: 1.7, 95% CI = 1.1–2.6
Woo Dae Kang	2011	Korea	204	49	IB-IIA	radical hysterectomy followed by adjuvant radiotherapy or primary radiotherapy with concurrent cisplatin-containing chemotherapy	28	176	NA	HR: 2.664, 95% CI = 1.437–4.938	NA
Shizhuo Wang	2012	China	24,041	NA	I-IV	NA	2082	21,959	NA	NA	HR: 1.704, 95% CI = 1.095–2.654

*HPV* Human papillomavirus, *DFS* Disease free survival, *HR* Hazard ratio, *CI* Confidence interval, *NA* Not available, *DFS* Disease free survival, *PFS* Progression free survival, *OS* Overall survival

**Table 3:** Study characteristics of studies investigating the prognostic relevance of HPV-16 and HPV-18

Author	Year	Country	Total number of patients	Mean age	Clinical stage	Treatment	Number of patients with HPV-16 positive	Number of patients with HPV-18 positive	Median DFS	Median PFS	Median OS
Mi Chen	2019	China	131	29–61	I-III	adjuvant radiation	88	19	HR: 1.13, 95% CI = 0.78–1.64	NA	HR: 1.39, 95% CI = 1.14–1.69
Yuanyuan Wang	2018	China	232	NA	I-III	adjuvant radiation	108	19	NA	Not significant	HR: 2.17, 95% CI = 1.20–3.92
Ruihong Lan	2017	China	40	40.21	II-III	adjuvant radiation	22	10	NA	NA	HR: 1.15, 95% CI = 1.01–1.31

## Results

### Study characteristics of the recruited studties

**Fig. 1:**
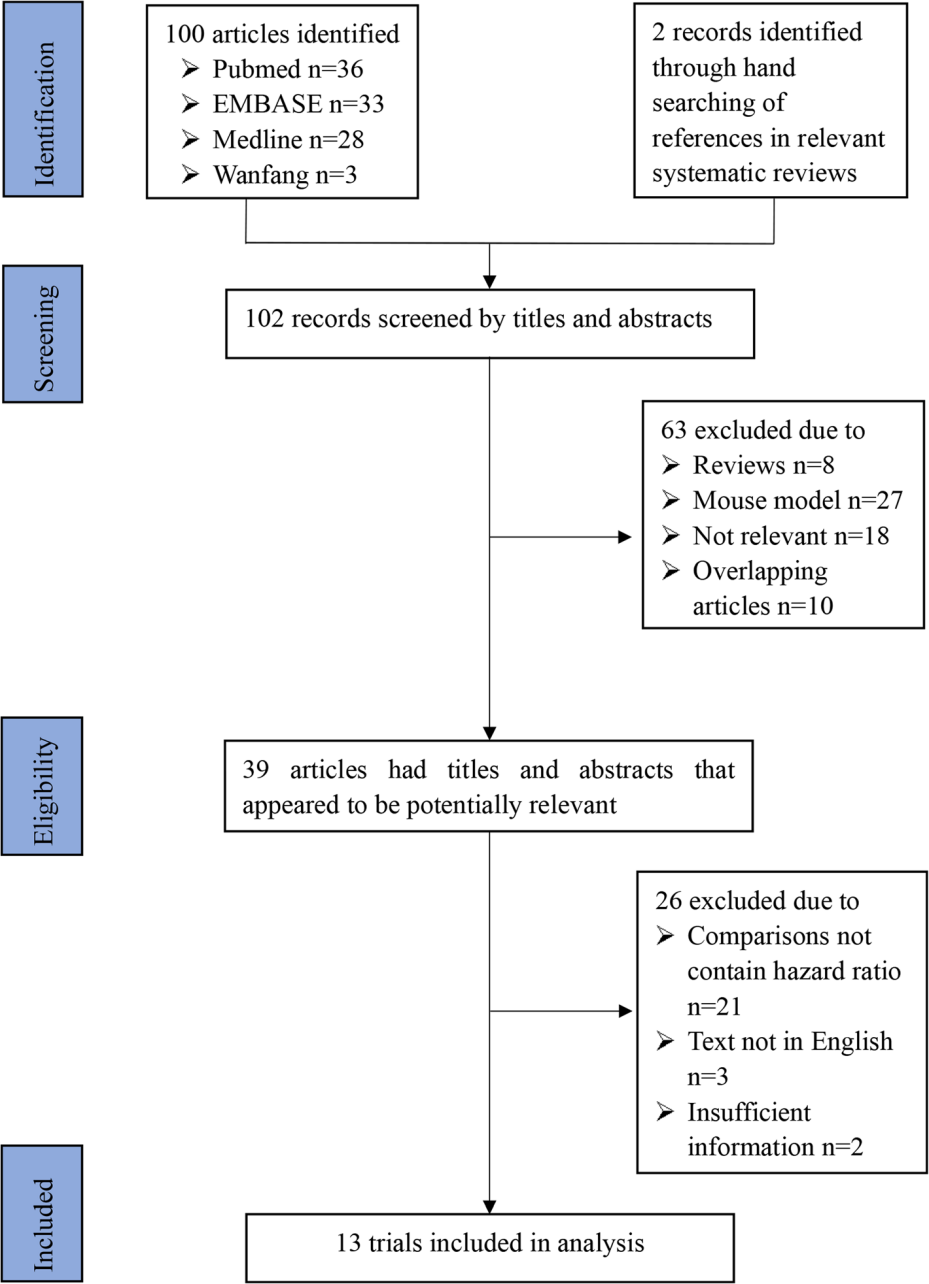
Flowchart of computerized search and the eligible studies included in this systematic review and meta-analysis

In total, 13 eligible studies were included in this systematic review and meta-analysis, with 6 trials about the survival data of cervical cancer patients infected with HPV 16 and 7 trials about the survival data of cervical cancer infected with HPV 18. Among these publications, 3 publications investigated the impact of both HPV 16 and HPV 18 on survival in cervical cancer patients. Three publications investigated the impact of both HPV 16 and HPV 18 on survival in cervical cancer patients. A flow chart of selection of the studies is illustrated in Fig. 1. Tables 1, 2 and 3 summarize the basic characteristics of the included studies of HPV 16 and HPV 18, including name of the first author, publication country, publication year, treatment, age of patients, clinical stage of tumor, number of patients infected with or without HPV, median DFS, median PFS and median OS. All 13 studies met the allocation concealment.

### Meta-analysis of survival outcome

#### Studies regarding the prognoctic relevance of HPV 16 on OS

**Fig. 2:**
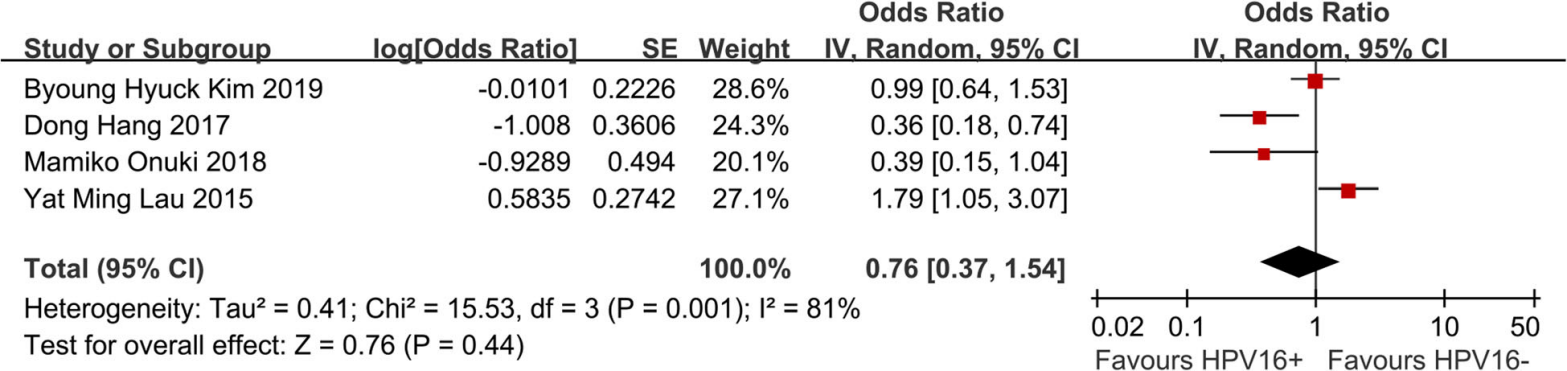
Meta analysis of impact of HPV 16 infection on OS in cervical cancer patients

We identified 4 eligible trials [4–7] including 977 cervical cancer patients, and investigated OS following HPV 16 positive versus HPV 16 negative patients. Our meta-analysis revealed that HPV 16 positive did not have any impact on OS (HR, 0.76; 95% CI = 0.37–1.54; *P* = 0.44, Fig. 2).

#### Studies regarding the prognoctic relevance of HPV 18 on OS

**Fig. 3:**
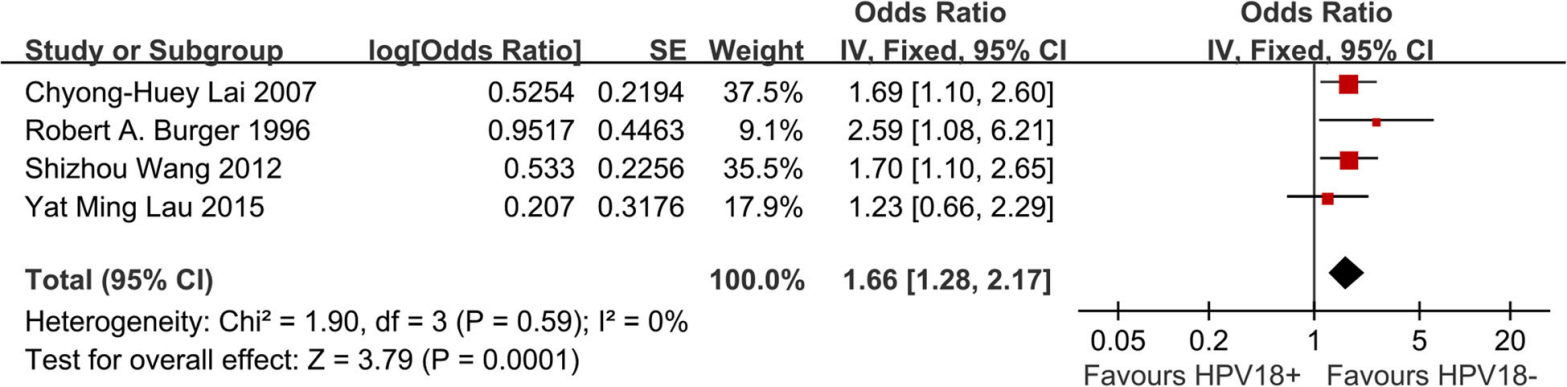
Meta analysis of impact of HPV 18 infection on OS in cervical cancer patients

We identified 4 eligible trials [4, 9, 11, 13] including 25,635 cervical cancer patients, and investigated OS following HPV 16 positive versus HPV 18 negative patients. Our meta-analysis revealed that cervical cancer patiensts infected with HPV 16 had worse OS compared with those not infected with HPV 18 (HR, 1.66; 95% CI = 1.28–2.17; *P* = 0.0001, Fig. 3).

#### Studies regarding the prognoctic relevance of HPV 18 on DFS

**Fig. 4:**
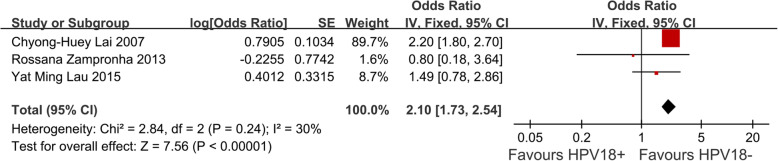
Meta analysis of impact of HPV 18 infection on DFS in cervical cancer patients

We identified 3 eligible trials [4, 9, 11] including 1389 cervical cancer patients, and investigated DFS following HPV 18 positive versus HPV 18 negative patients. Our meta-analysis revealed that cervical cancer patiensts infected with HPV 18 had worse DFS compared with those not infected with HPV 18 (HR, 2.10; 95% CI = 1.73–2.54; *P* < 0.0001, Fig. 4).

#### Studies regarding the prognoctic relevance of HPV 18 on PFS

**Fig. 5:**
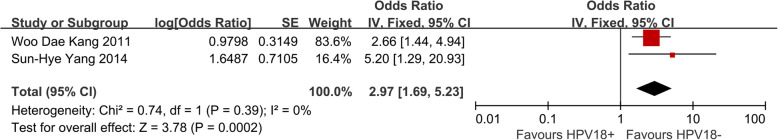
Meta analysis of impact of HPV 18 infection on PFS in cervical cancer patients

We identified 2 eligible trials [8, 12] including 320 cervical cancer patients, and investigated PFS following HPV 18 positive versus HPV 18 negative patients. Our meta-analysis revealed that cervical cancer patiensts infected with HPV 18 had worse PFS compared with those not infected with HPV 18 (HR, 2.97; 95% CI = 1.69–5.23; *P* = 0.00012, Fig. 5).

#### Studies regarding the prognoctic relevance of HPV 18 in comparision with HPV 16

**Fig. 6:**
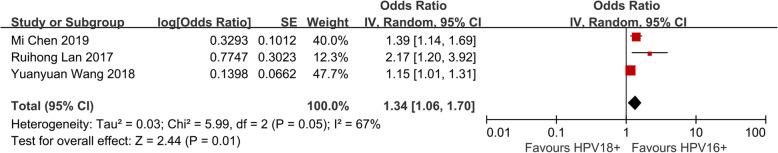
Meta analysis of impact of HPV 18 in comparision with HPV 16 infection on OS in cervical cancer patients

We identified 3 eligible trials [20–22] including 403 cervical cancer patients, and investigated OS following HPV 18 positive versus HPV 16 positive patients. Our meta-analysis revealed that cervical cancer patiensts infected with HPV 18 had worse PFS compared with those infected with HPV 16 (HR, 1.34; 95% CI = 1.06–1.70; *P* = 0.01, Fig. 6).

### Publication bias

No evidence of publication bias was found in our study by funnel plot, Egger’s test (*P* > 0.05) and Begg’s test (*P* > 0.05).

## Discussion

Our study was the first systematic review and meta analysis investigating the impact of HPV 16 and HPV 18 on survival in cervical cancer patients. Our results indicated that patients infected with HPV 18 had worse DFS, PFS and OS compared with cervical cancer patients without HPV 18 infection. While the infection of HPV 16 had no impact on survival in cervical cancer patients compared with all other patients. Cervical cancer patients with HPV 18 infection had worse OS in comparision with patients with HPV 16 infection.

An ongoing HPV infection could be a risk factor of infection of another HPV genotype [23, 24] and infection of multiple genotypes of HPV could be a risk factor of persistent infection [25, 26], which is foundermental in development of cervical leisions into cervical cancer. Previous study of 43 cervical cancer patients found that an infection of HPV 18 could be related to lack of treatment response [27]. However, previous published studies regarding the unfavorable prognosis of HPV genotypes did not reach consistency.

During the development and progression of cervical cancer, patients could be infected with many genotypes of HPV, including high, intermediate and low risk HPV. However, more publications indicating the negative impact of HPV 16 or HPV 18 on survival in cervical cancer patients [28, 29]. These studies suggested that HPV to be a prognostic indicator for survival in cervical cancer patients and is of significance identifying specific therapies against HPV-harboring cervical cancer patients. However, some of these studies had only small sample size and did not adjust the prognostic relavance of HPV 16 or HPV 18 using a multivariate cox regression model. Also, studies did not reach consistency as several studies found that HPV 16 or HPV 18 did not have impact on survival. Our study summarizd all the published articles about the HPV 16 and HPV 18 as a prognostic parameter for survival in cervical cancer patients and found that only HPV 18 was negatively associated with survival (OS: HR, 1.66; 95% CI = 1.28–2.17; *P* = 0.0001, Fig. 3; DFS: HR, 2.10; 95% CI = 1.73–2.54; *P* < 0.0001, Fig. 4; PFS: HR, 2.97; 95% CI = 1.69–5.23; *P* = 0.00012, Fig. 5). Patients with HPV 18 infection had worse OS compared with patients with HPV 16 infection (HR, 1.34; 95% CI = 1.06–1.70; *P* = 0.01, Fig. 6).

Awareness of the HPV 16 or 18 infection should be raised when it can be controlled during the disease progression. However, for HPV infection, that is not the case. There is no specific medicines to treat HPV infection. The good news is that patients’ immune system could clear 90% of the HPV infection within 2 years, as long as people with normal immunity [30]. For these patients infected with high risk HPV, the immune function is important for cervical carcinogenesis [31]. Chronic stromal inflammation and immune deviation may eventually determine the progression of cervical cancer [31]. Understanding the mechanisms of the HPV infections may help to define new tools for better treatment required to efficiently combat cervical cancer.

We must admit that our meta analysis has several limitations. Firstly, some of the studies recruited in our meta analysis did not have a large sample size, which would ruin the power of statistics. Secondly, the prognostic relavance of HPV 16 or 18 in some studies are not validated using a multivariate analysis considering the impact of confounding factors, such as tumor size, clinical stage and metastasis. Thirdly, these studies recruited a broad spectrum of patients, range from stage I to stage IV patients, which could raise the heterogeneity of the meta analysis. We were not able to get more data about patients with multiple infection of HPV genotypes, such as treatment. Treatment has an impact on the HPV infection, for example, HPV 18 is more resistant to radiotherapy. There are not many studies published comparing the survival relevance of HPV 16 to HPV 18, thus we were not able to make subgroup analyses based on different treatment, such as adjuvant chemotherapy or radiotherapy. We summarized the treatment methods in Table 1 to Table 3 and all the studies recruited in Table 3 using the adjuvant radiation, thus it is not necessary to make subgroup analysis for meta analysis of impact of HPV 16 in comparision with HPV 18 in cervical cancer patients. In addition, our study was the first to gain the importance of recognizing the HPV infection during disease progress and raised the awareness of its prognostic relevance. Large scale clinical trials evaluating the impact of HPV 18 versus HPV 16 on survival in cervical cancer patients in different disease stages under various treatment methods such as adjuvant chemotherapy or radiotherapy are needed.

## Conclusion

Cervical cancer patients infected with HPV 18 had worse survival in comparision to patients with HPV 16 infection.

## Data Availability

Data are available from the corresponding author on reasonable request.
